# Dissociable Processes of Cognitive Control during Error and Non-Error Conflicts: A Study of the Stop Signal Task

**DOI:** 10.1371/journal.pone.0013155

**Published:** 2010-10-06

**Authors:** Olivia M. Hendrick, Jaime S. Ide, Xi Luo, Chiang-shan R. Li

**Affiliations:** 1 Interdepartmental Neuroscience Program, Yale University School of Medicine, New Haven, Connecticut, United States of America; 2 Department of Psychiatry, Yale University School of Medicine, New Haven, Connecticut, United States of America; 3 Department of Statistics, Yale University School of Medicine, New Haven, Connecticut, United States of America; 4 Department of Neurobiology, Yale University School of Medicine, New Haven, Connecticut, United States of America; Kyushu University, Japan

## Abstract

**Background:**

Conflict detection and subsequent behavioral adjustment are critical to daily life, and how this process is controlled has been increasingly of interest. A medial cortical region which includes the anterior cingulate cortex (ACC) has been theorized to act as a conflict detector that can direct prefrontal activity for behavioral adjustments. This conflict monitoring hypothesis was supported by many imaging studies of the Stroop task, with a focus on non-error processes. Here we sought to examine whether this circuit could be generalized to the stop signal task (SST), another behavioral paradigm widely used to study cognitive control. In particular, with a procedure to elicit errors in the SST, we examined whether error and non-error control were mediated by the same pathways.

**Methodology/Principal Findings:**

In functional magnetic resonance imaging of 60 healthy adults, we demonstrated that the medial cortical activity during stop success (SS) as compared to go success (G) trials is correlated with increased prefrontal activity in post-stop SS as compared to post-go SS trials, though this correlation was not specific to the medial cortical region. Furthermore, thalamic and insular rather than medial cortical activation during stop error (SE) as compared to G trials correlated with increased prefrontal activity in post-stop SS as compared to post-go SS trials.

**Conclusions/Significance:**

Taken together, these new findings challenge a specific role of the ACC and support distinct pathways for error and non-error conflict processing in cognitive control.

## Introduction

Cognitive control is critical to learning and survival in a constantly changing environment. Understanding the neural processes underlying cognitive control has been of increasing interest among neuroscientists. A key component process of cognitive control is the detection of conflict. An error or non-error conflict is prone to occur when multiple sources of information demand different and oftentimes opposing responses, such as when one is required to report the color of the ink in which a color word (RED) is printed. In a Stroop task – one of most commonly used behavioral tasks to study cognitive control – participants are required to do so for many color words, most of which are congruent because the word is printed in the same color while others are incongruent because the word is printed in a color different from that specified by the word. Participants invariably take longer to respond to an incongruent compared to a congruent word because the former involves a response engaged by the rule that is in conflict with a response evoked by linguistic tendency.

An influential theory of how our brain implements cognitive control is the conflict monitoring hypothesis. This hypothesis proposes that the anterior cingulate cortex (ACC) detects conflict and then engages the prefrontal cortex to “control” or better respond to any future conflicts [1]. Numerous fMRI studies confirmed the role of ACC in conflict detection [2]–[5] and many also linked activation of ACC to performance monitoring during cognitive control [6]–[7]. For instance, using the Stroop task, Kerns and colleagues [7] separated trials by whether they were congruent or incongruent and, in addition, whether they followed a congruent or incongruent trial. They observed greater ACC activation during incongruent than congruent trials. Furthermore, by comparing activity between incongruent trials that followed incongruent trials (iI) and those that followed congruent trials (cI), they observed greater activation in the prefrontal cortex during iI trials as compared to cI trials. Importantly, these investigators found that the prefrontal cortical activation correlated with the extent to which the ACC had been activated on the previous trial, in support of the conflict monitoring hypothesis.

Past fMRI work suggested that error and non-error conflicts are dissociable as they involve different regional brain activations [8]–[10]. The thalamus, for instance, seems to differentiate between error and non-error conflicts [3], [10]–[11]. However, there is little information about whether error and non-error conflict involve different neural processes in cognitive control, perhaps because participants generally make very few errors in the Stroop task. Our previous fMRI studies attempted to address this issue by employing the stop signal task (SST), in which a staircase procedure was used to elicit errors [10], [12]. Following an error, subjects tended to respond with a longer latency on the subsequent “go” trial, a phenomena known as post-error slowing (PES). We observed robust error-related activation in the dorsal ACC and activation in the ventral lateral prefrontal cortex during PES [10], [12]. However, this prefrontal activity during PES did not correlate to error-related activity in the ACC, a finding that appeared to be inconsistent with the conflict monitoring theory.

This study sought to further pursue these error-related findings as well as to examine the conflict monitoring theory. We hypothesized that error and non-error conflict would involve different neural processes during cognitive control. Using the SST, we compared stop (incongruent) with go (congruent) trials to examine conflict processing and compared stop success trials preceded by stop and go trials to examine post-conflict control, emulating previous studies of the Stroop task. We then explored whether error and non-error conflicts engage different regional brain processes in cognitive control by correlating the activity of the conflict areas during stop success or stop error trials to activity in the “control” regions. Note that, unlike our previous work examining post-error slowing [12], which was a quantifiable behavioral change, a stop success trial did not involve a reaction time. Thus, the current work built on an assumption of greater post-conflict control in the post-stop stop success as compared to post-go stop success trials.

## Methods

### Subjects and behavioral task

Sixty healthy adults (30 males, 22–42 years of age, all right-handed and using their right hand to respond) were compensated for their participation in the study. All subjects signed a written consent, in accordance to a protocol approved by the Yale Human Investigation Committee.

We employed a simple reaction time task in this stop-signal paradigm [10], [12]–[15]. There were two trial types: “go” and “stop,” randomly intermixed. A small dot appeared on the screen to engage attention at the beginning of a go trial. After a randomized time interval (fore-period) between 1 and 5 s, the dot turned into a circle (the “go” signal), prompting the subjects to quickly press a button. The circle vanished at a button press or after 1 s had elapsed, whichever came first, and the trial terminated. A premature button press prior to the appearance of the circle also terminated the trial. Three quarters of all trials were go trials. The remaining one quarter were stop trials. In a stop trial, an additional “X,” the “stop” signal, appeared after and replaced the go signal. The subjects were told to withhold button press upon seeing the stop signal. Likewise, a trial terminated at button press or when 1 s had elapsed since the appearance of the stop signal. The stop signal delay (SSD) – the time interval between the go and stop signal – started at 200 ms and varied from one stop trial to the next according to a staircase procedure: if the subject succeeded in withholding the response, the SSD increased by 64 ms; conversely, if they failed, SSD decreased by 64 ms [16]–[17]. There was an inter-trial-interval of 2 s. Subjects were instructed to respond to the go signal quickly while keeping in mind that a stop signal could come up in a small number of trials. Prior to the fMRI study each subject had a practice session outside the scanner for approximately 10 minutes, to ensure they fully understood the task. In the scanner each subject completed four 10-min runs of the task. Depending on the actual stimulus timing (trials varied in fore-period duration) and speed of response, the total number of trials varied slightly across subjects in an experiment. With the staircase procedure we anticipated that the subjects would succeed in withholding their response in approximately half of the stop trials.

The stop signal reaction time (SSRT) was calculated by subtracting the critical SSD, or the estimated SSD at which 50% of stop trials were correct, from the median go RT. We also derived a measure of post-error slowing (PES), as an index of general performance monitoring, by computing the effect size of the difference between the RT of post-stop error and post-go go trials [12].

### Imaging protocol

Conventional T_1_-weighted spin echo sagittal anatomical images were acquired for slice localization using a 3T scanner (Siemens Trio). Anatomical images of the functional slice locations were next obtained with spin echo imaging in the axial plane parallel to the AC-PC line with TR  = 300 ms, TE  = 2.5 ms, bandwidth  = 300 Hz/pixel, flip angle  = 60°, field of view  = 220×220 mm, matrix  = 256×256, 32 slices with slice thickness  = 4 mm and no gap. Functional, blood oxygenation level dependent (BOLD) signals were then acquired with a single-shot gradient echo echo-planar imaging (EPI) sequence. Thirty-two axial slices parallel to the AC-PC line covering the whole brain were acquired with TR  = 2,000 ms, TE  = 25 ms, bandwidth  = 2004 Hz/pixel, flip angle  = 85°, field of view  = 220×220 mm, matrix  = 64×64, 32 slices with slice thickness  = 4 mm and no gap. Three hundred images were acquired in each run for a total of four runs.

### Data analysis and statistics

Data were analyzed with Statistical Parametric Mapping (SPM5, Wellcome Department of Imaging Neuroscience, University College London, U.K.). Images from the first five TRs at the beginning of each trial were discarded to enable the signal to achieve steady-state equilibrium between RF pulsing and relaxation. Images of each individual subject were first corrected for slice timing and realigned (motion-corrected). A mean functional image volume was constructed for each subject for each run from the realigned image volumes. These mean images were normalized to an MNI (Montreal Neurological Institute) EPI template with affine registration followed by nonlinear transformation [18]–[19]. The normalization parameters determined for the mean functional volume were then applied to the corresponding functional image volumes for each subject. Finally, images were smoothed with a Gaussian kernel of 10 mm at Full Width at Half Maximum.

**Figure 1:**
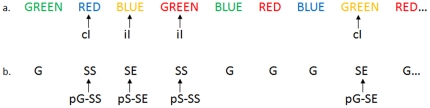
Trial structure of the stop signal and Stroop task. Example of a trial sequence in stop signal task (b) that parallels that in the Stroop task (a). In the Stroop task, color words that are printed in a color different from what the word says represent incongruent (I) trials; otherwise, they are congruent (C) trials. In the stop signal task, both stop success (SS) and stop error (SE) trials are incongruent trials, and involved in conflict processing, as compared to go (G) trials, which are congruent trials. In both tasks, trials are distinguished by their preceding trials. Thus, an incongruent trial following a congruent trial is “cI” in the Stroop task, and a SS trial following a go trial is “pG-SS” in the stop signal task.

Four main types of trial outcome were first distinguished: go success (G), go error (F), stop success (SS), and stop error (SE) trial. An SS or SE trial involves incongruent goals between the prepotency to respond and the motor intention to withhold the response, and thus is “high-conflict,” compared to a G trial. SS and SE trials were further defined by whether they followed a stop (pS) or a go (pG) trial. This was homologous to the iI and cI trials in the Stroop task (**Fig. 1**). A single statistical analytical design was constructed for each individual subject, using the general linear model (GLM) with the onsets of go signal in each of these trial types convolved with a canonical hemodynamic response function (HRF) and with the temporal derivative of the canonical HRF and entered as regressors in the model [20]. Realignment parameters in all 6 dimensions were also entered in the model. The data were high-pass filtered (1/128 Hz cutoff) to remove low-frequency signal drifts. Serial autocorrelation of the time series violated the GLM assumption of the independence of the error term and was corrected by a first-degree autoregressive or AR (1) model [21]. The GLM estimated the component of variance that could be explained by each of the regressors.

The *con* or contrast (difference in β) images of the first-level analysis were used for the second-level group statistics [22]. Brain regions were identified using an atlas [23]. All templates are in Montreal Neurological Institute (MNI) space and voxel activations are presented in MNI coordinates. We used MarsBaR to derive for each individual subject the effect size of activity change for regions of interest [24]; http://marsbar.sourceforge.net/.

### Mediation Analysis

Mediation analyses were performed to further characterize the functional connectivity between the regions of interest [25], using the toolbox M3, developed by Tor Wager and Martin A. Lindquist (http://www.columbia.edu/cu/psychology/tor/). Mediation analyses are widely used in social and economic research to examine whether a relationship between two variables is mediated by an intervening variable [26]–[27]. It was also applied recently to fMRI data analysis [28]. In a mediation analysis, relation between the independent variable X and the dependent variable Y, i.e. X→Y, is tested to see if it is significantly mediated by a variable M. The mediation test is performed by employing three regression equations [25]:
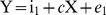


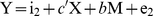


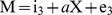
where *a* represents X→M, *b* represents M→Y (controlling for X), *c*' represents X→Y (controlling for M), and *c* represents X→Y. i_1_, i_2_ and i_3_ are the intercepts, and e_1_, e_2_ and e_3_ are the residuals. In the literature, *a*, *b*, *c* and *c*' were referred as *path coefficients* or simply *paths* [25], [28], and we followed this notation. Variable M is said to be a mediator of X→Y, if (*c*–*c*') is significantly different from zero, which is mathematically equivalent to the product of the paths a*b [25]. If the product a*b and also the paths *a* and *b* are significant, one concludes that X→Y is mediated by M. Notice that path *b* is the relation between Y and M, controlling for X, and should not be confused with the linear correlation between Y and M.

## Results

### Behavioral performance

The subjects succeeded in an average of 95.9±4.3% (mean ± standard deviation) of go trials and 50.6±2.5% of stop trials, suggesting that the staircase procedure was adequately tracking their performance. The median go trial reaction time was 568±127 ms and the stop signal reaction time was 205±39 ms. The effect size of post-error slowing was 1.65±1.62.

### Conflict and post-conflict regional brain activations

**Figure 2:**
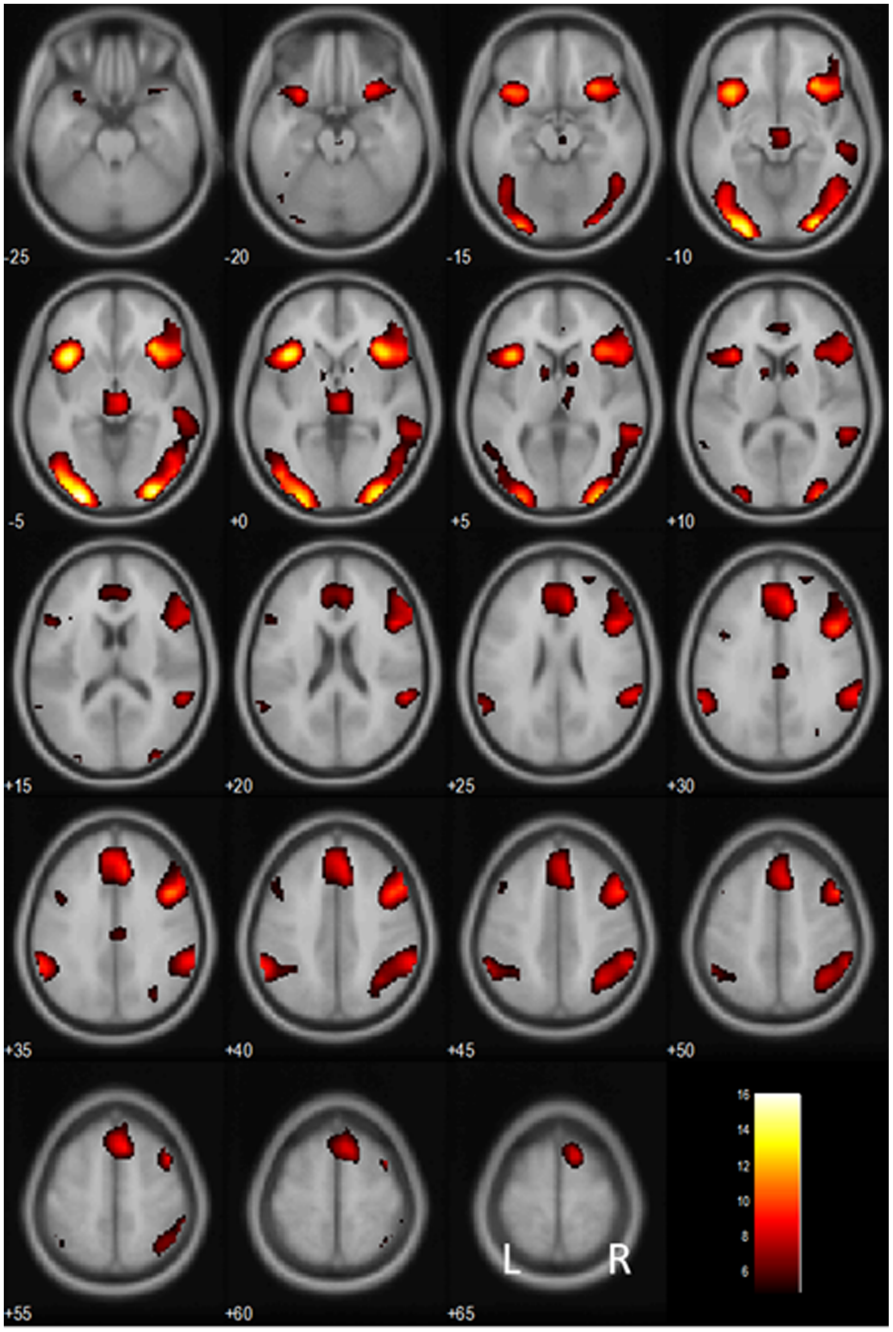
Brain regions showing more activation in stop as compared with go trials. BOLD contrasts are superimposed on a T1 structural image in axial sections from *z* = −25 to *z* = 65. The adjacent sections are 5 mm apart. The color bar represents voxel T value. L, Left; R, Right.

At a threshold of p<0.05, corrected for family-wise error (FWE) of multiple comparisons, we identified brain regions showing greater activation during stop as compared to go trials, including the anterior cingulate cortex (ACC)/supplementary motor area (SMA) including the preSMA, lateral frontal cortices, bilateral inferior parietal cortices and temporal parietal junction, visual cortices, thalamus including the epithalamus and part of the midbrain, and caudate head (**Fig. 2**; **Table 1**).

**Table 1:** Brain regions more activated in stop as compared with go trials.

		MNI Coordinates (mm)				
Cluster Size (voxels)	Voxel Z Value	X	Y	Z	Side	Identified Region
110	7.14	4	−20	−4	R/L	thalamus
	4.65	4	−24	−20	R/L	midbrain
29	5.6	4	−24	32	R/L	cingulate G
522	7.47	8	28	32	R	anterior cingulate G/S
	7.3	8	20	56	R	anterior cingulate G/S; SMA, preSMA
	7.05	−4	40	32	L	anterior cingulate G/S
19	5.9	12	8	8	R	caudate head
781	Inf	32	24	−4	R	insula
	Inf	44	12	36	R	inferior frontal G
	7.68	48	12	52	R	middle frontal G
842	Inf	32	−92	−8	R	G descendens (occipital cortex)
	7.84	44	−80	−8	R	middle occipital G
	7.19	60	−44	36	R	supramarginal G
18	5.68	−12	4	8	L	caudate head
521	Inf	−28	−96	−8	L	G descendens (occipital cortex)
	7.47	−60	−48	36	L	supramarginal G
	7.04	−40	−64	−12	L	middle occipital G
284	Inf	−40	16	−4	L	insula

Statistical threshold: *p<0.005*, *uncorrected*; *extent*, *5 voxels*. *G*, *Gyrus*; *S*, *Sulcus*; *L*, *left*; *R*, *right*; *SMA*, *supplementary motor area*. All peak activations greater than 8 mm apart are identified.

**Figure 3:**
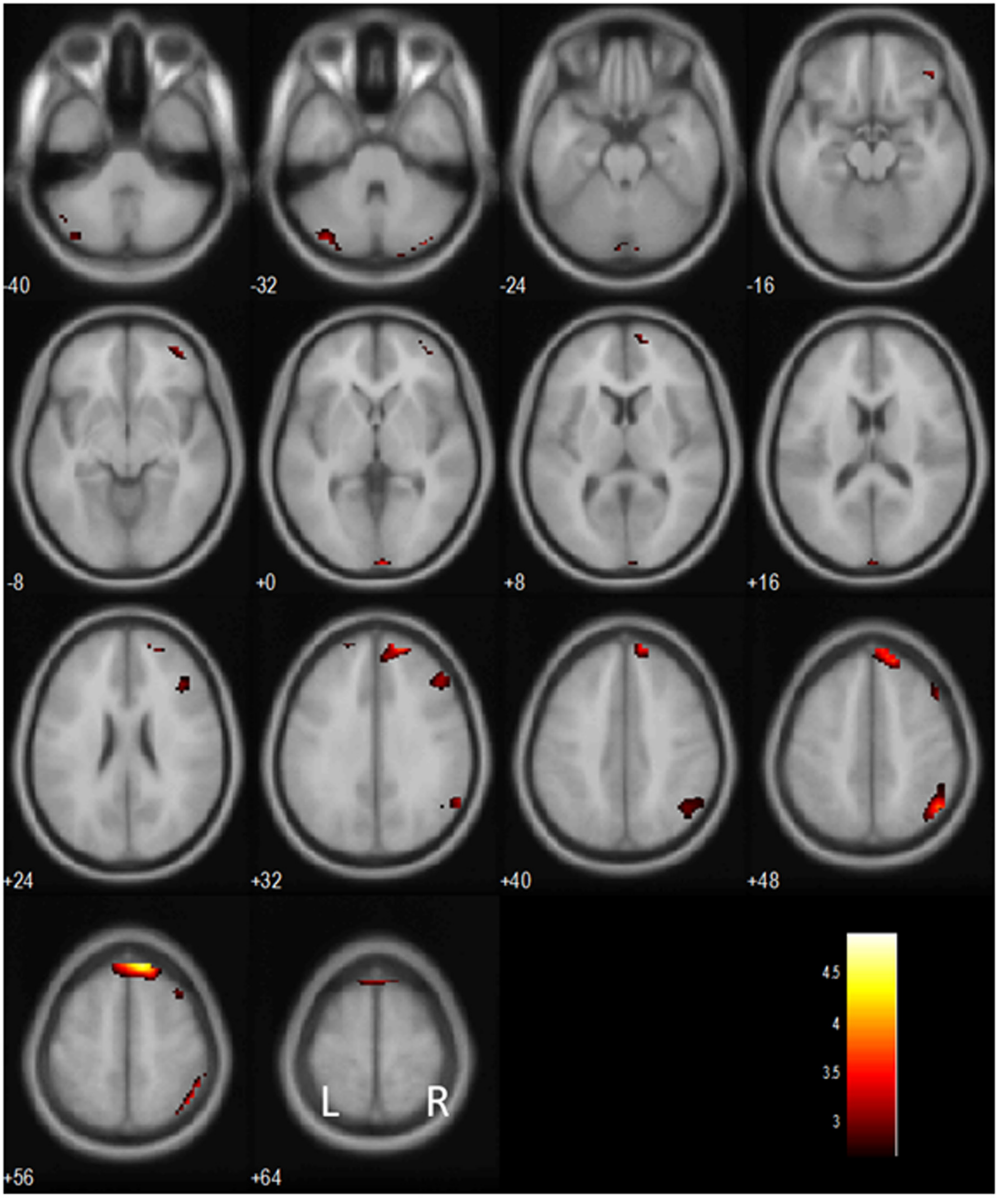
Brain regions showing more activation in post- stop as compared with post-go stop success trials. BOLD contrasts are superimposed on a T1 structural image in axial sections from *z* = −40 to *z* = 64. The adjacent sections are 8 mm apart. The color bar represents voxel T value. L, Left; R, Right.

We compared post-stop and post-go stop success (pS-SS and pG-SS, respectively) trials to examine regional processes of post-conflict control, following previous studies of the Stroop task. For pS-SS trials, the first stop included both error and success trials as there were not enough of either to consider separating the two in GLM analyses. At a threshold of p<0.005, uncorrected, and 5 voxels in the extent of activation, this contrast (pS-SS > pG-SS) involved activation of several prefrontal structures, including the right lateral orbitofrontal cortex (OFC), bilateral lateral prefrontal cortices, and right inferior parietal cortices, as well as distinct clusters in the cerebellum (**Fig. 3**; **Table 2**).

**Table 2:** Brain regions more activated in post-stop as compared with post-go stop success trials.

		MNI Coordinates (mm)				
Cluster Size (voxels)	Voxel Z Value	X	Y	Z	Side	Identified Region
14	3.28	4	−100	0	L	superior occipital G
140	4.48	12	36	60	L	superior frontal G
	3.64	8	48	52	L	superior frontal G
	3.49	16	52	32	L	superior frontal G
8	2.95	12	64	8	L	superior frontal G
15	3.03	36	56	−4	R	lateral orbital G
31	3.13	44	28	28	R	middle frontal G
15	3.25	48	20	48	R	middle frontal G
	2.88	40	20	56	R	middle frontal G
32	3.34	−48	−68	−36	L	cerebellar lobule
7	3.37	36	−84	−32	R	cerebellar lobule
90	3.83	48	−60	52	R	angular G
	3.15	52	−48	56	R	supramarginal G
	3.04	60	−56	32	R	angular G

Statistical threshold: *p<0.005*, *uncorrected*; *extent*, *5 voxels*. *G*, *Gyrus*; *S*, *Sulcus*; *L*, *left*; *R*, *right*. All peak activations greater than 8 mm apart are identified.

### Linear correlation between conflict and post-conflict processes

We derived the effect sizes of conflict-related activation of each of the brain regions (Fig. 2; Table 1) separately for stop success (SS) and stop error (SE) trials; i.e., SS >G and SE >G, respectively, and correlated these measures to the effect sizes of post-conflict activity changes: pS-SS >pG-SS (Fig. 3; Table 2), using Pearson's regression. The regions of interest (ROIs) used in the correlation analyses were composed of the spatially contiguous voxels of the activations clusters. **Table 3a** lists the regression coefficient (R) for these pair-wise linear regressions and highlights those that are significant (p<0.005). The results showed that many brain regions including the dorsal anterior cinguate cortex (dACC)/supplementary motor area (SMA) as well as the visual cortices and parietal regions demonstrated non-error conflict activity (SS>G) that is correlated with control activity (pS-SS>pG-SS) in the prefrontal cortices. These correlations were particularly strong to the right lateral prefrontal and orbitofrontal regions. In contrast, error conflict (SE>G) activity of the thalamus and insula showed strongest correlations to the prefrontal activation during post-conflict control (**Table 3b**).

**Table 3:** R-values of correlations between post-stop as compared with post-go stop success regions and stop success (a) or stop error (b) as compared with go trials.

						S>G regions (SS>G)					
		Thal	Cing	ACC-SMA	R Caud	R LPFC	R Vis	L Caud	L Vis	L Insul	L IPC
	L Occ	0.064	−0.004	0.136	0.155	0.165	0.086	0.137	0.178	0.073	−0.076
	L FPC	−0.048	0.154	0.314	0.179	0.384	0.323	0.112	0.452#	0.287	0.183
	L FPC	0.013	0.024	0.206	0.050	0.256	0.269	−0.012	0.374	0.283	0.299
	R OFC	0.190	0.385	0.530*	0.394∧	0.577*	0.614*	0.378	0.585*	0.604*	0.558*
	CBL	−0.005	0.167	−0.046	−0.026	−0.001	−0.095	−0.012	0.005	−0.088	−0.199
	IFC	0.119	0.368	0.386	0.260	0.510*	0.474#	0.239	0.368	0.541*	0.466#
	R LPFC	0.111	0.300	0.529*	0.479#	0.579*	0.556*	0.466#	0.494#	0.544*	0.513*
	R PPC/Occ	0.130	0.453#	0.489#	0.380	0.576*	0.637*	0.355	0.532*	0.567*	0.609*

						S>G regions (SE>G)					
		Thal	Cing	ACC-SMA	R Caud	R LPFC	R Vis	L Caud	L Vis	L Insul	L IPC
	Occ	0.198	−0.203	0.003	0.078	0.120	0.008	0.003	0.134	0.099	−0.318
	R FPC	0.366	−0.108	0.017	0.213	0.191	0.148	0.075	0.352	0.187	−0.223
	L FPC	0.395∧	0.098	0.224	0.247	0.205	0.222	0.149	0.272	0.218	0.030
	R OFC	0.491#	0.173	0.191	0.270	0.290	0.268	0.173	0.354	0.420∧	0.058
	CBL	0.155	−0.109	0.134	0.212	0.182	0.109	0.177	0.117	0.105	−0.108
	IFC	0.190	0.167	0.032	0.098	0.161	0.163	0.038	0.270	0.205	0.002
	R LPFC	0.436∧	0.282	0.260	0.249	0.335	0.271	0.204	0.365	0.463#	0.043
	PPC/Occ	0.405∧	0.272	0.103	0.249	0.257	0.277	0.170	0.378	0.345	0.029

∧ p<0.005.

# p<0.001.

* p<0.0001.

*Thal, Thalamus; Cing, Cingulate; Caud, Caudate; Vis, Visual; Insul, Insula; FPC, Frontopolar Cortex; Occ, Occipital; OFC, Orbitofrontal Cortex; CBL, Cerebellum; IFC, Inferior Frontal Cortex; PPC, Posterior Parietal Cortex; IPC, Inferior Parietal Cortex; L, left; R, right.*

### Mediation Analysis

Many brain regions other than the ACC/SMA showed conflict activities that were correlated with post-conflict prefrontal activations. Although this finding appeared to be at odds with a specific role of the medial cortical region in cognitive control, one needs to rule out the possibility that the correlations observed for these other brain regions were mediated by the ACC/SMA. We thus focused on the right lateral PFC (LPFC), a post-conflict “control” region and examined whether the conflict activities of the left inferior parietal cortex (IPC), left insula (Insul), and right visual (Vis) were mediated by the ACC/SMA. The results showed that there were not significant mediations between the conflict regions and the right LPFC by the ACC/SMA. That is, none of the mediation paths a*b were significant (**Table 4**).

**Table 4:** Mediation analysis results between the conflict areas: right visual (R Vis), left insula (L Insul), and left inferior parietal cortex (L IPC), and the post-conflict region right lateral prefrontal cortex (R LPFC) with the potential mediator ACC/SMA.

R Vis→R LPFC mediated by ACC/SMA			
	*a* Path	*b* Path	*a* * *b* Mediation path
**β**	1.21	0.25	0.31
**p-values**	0.0001*	0.1964	0.1549

L Insul→R LPFC mediated by ACC/SMA			
	*a* Path	*b* Path	*a* * *b* Mediation path
**β**	1.31	0.23	0.31
**p-values**	0.0003*	0.3928	0.3817

L IPC→R LPFC mediated by ACC/SMA			
	*a* Path	*b* Path	*a* * *b* Mediation path
**β**	0.85	0.34	0.3
**p-values**	0.001*	0.1703	0.1894

*β* denotes the regression coefficients and p-values are uncorrected.

*indicates significant connections at p<0.0125, Bonferroni corrected.

## Discussion

There are two main findings in the current study. First, non-error conflict activations of the medial frontal cortex including the ACC correlated with post-conflict prefrontal activations but this was not true of error conflicts. The correlation between the medial frontal cortex and post-conflict prefrontal activations was not specific as many other brain regions that exhibited activation during non-error conflicts also correlated with post-conflict prefrontal activations. Second, thalamic and insular activation during error but not non-error conflicts correlated with post-conflict prefrontal activations. Taken together, these results suggested dissociable neural pathways for cognitive control during error and non-error conflicts.

### Error and non-error conflict control

In the SST, stop trials involve conflict between a pre-potent go response and a stop signal demanding withdrawal of the response. Thus, compared to go trials, stop trials engaged conflict processing, which, according to the conflict monitoring theory, facilitates cognitive control during subsequent trials in the prefrontal cortex [1], [29]–[32]. Previous event related potential (ERP) studies dissociated error from non-error conflicts. Compared to successful high-conflict trials, error trials resulted in an ERP with greater negativity (ERN) followed by positivity (Pe), timed locked to the motor response [33]–[36]. Investigators have attempted to identify the source of ERN but to our knowledge produced inconsistent results. Some but not all studies localized the generator of the ERN to the ACC [37]–[40]. It was also unclear whether the ERN is associated with post-error behavioral adjustment; some studies have reported a lack of association between ERN and post-error behavioral adjustment [41]–[43], while others have found the opposite [37], [44]–[45]. If the ERN originated in the dorsal ACC, one would speculate that the ERN would not correlate with post-error behavioral or neural measures, on the basis of the current findings.

The current results suggested that thalamus mediates error-related post-conflict control, in accord with our recent functional connectivity study that described a thalamo-cortical circuit during post-error slowing [46]. Many preclinical and clinical studies implicated thalamus in performance monitoring, such as in matching sensory feedback with expected outcome of a motor response [47]–[48], re-evaluation of a reinforcer [49], task planning on the basis of external information [50], processing corollary discharge of an eye movement [51]–[52], as well as reception of negative feedback during the Wisconsin Card Sorting Task [53]. Anatomical studies have consistently established a link between the mediodorsal thalamus and prefrontal cortices in humans as well as non-human primates [54]–[56]. The insula responded to errors in a wide variety of behavioral tasks [3], [5], [57]–[62]. This activation may reflect a heightened autonomic arousal or affective response to errors [63]–[65] or awareness of as well as attentional orientation to errors [61]–[62], [66]. Thus, the current study extended these previous findings by specifying a link between error conflict thalamic and insular activity and prefrontal activity during post-conflict control.

### Anterior cingulate cortex (ACC) and cognitive control

The current findings provide limited support for the conflict-monitoring hypothesis. The ACC showed greater activation during stop as compared to go trials and the effect size of activation was correlated with prefrontal activation during post-conflict processing. On the other hand, the ACC was not the only conflict-processing brain region that influenced post-conflict prefrontal activity. Furthermore, the results of mediation analyses indicated that these other conflict-processing brain regions likely do not influence post-conflict lateral prefrontal activity via the ACC/SMA.

Earlier studies have presented results that are not explained by the conflict-monitoring hypothesis [67]–[73]. For instance, lesion studies do not support an indispensable role of ACC in cognitive control; Fellows and Farah showed that patients with ACC damage exhibited normal adjustment in performance following manipulation of response conflict in both Stroop and go-no go tasks [70], a finding that was further confirmed in a more recent study using the flanker task [36]. Other lesion studies with humans also showed that even when a lesion decimates the ACC, subjects can still perform cognitive control tasks at control levels, including demonstration of post-error slowing [67]–[70]. If the ACC were the only region to activate the cognitive control network, we would expect more severe behavioral effects in these populations. Additionally, Mansouri and colleagues [74] created lesions in the ACC or dorsolateral prefrontal cortex (DLPFC) of monkeys and had them perform a modified Wisconsin card sorting task that allowed post-conflict behavior to be monitored. They observed that conflict-induced behavioral adjustment persisted after lesions within the ACC but disappeared after lesions within the DLPFC. Furthermore, in different monkeys performing the same task, neuronal activities recorded from the DLPFC but not ACC responded to conflict either in current or previous trials. These findings suggest that information about conflict is not necessarily processed in the ACC, but in at least the DLPFC.

Other studies in monkeys have found behavioral effects of conflict without corresponding modulation of neuronal activity in the ACC [4], [75]. For instance, Nakamura, Roesch, & Olson [75] had monkeys perform a saccade-countermanding task. The low conflict condition was when the cue's location also indicated the direction of the correct saccade, while the reverse was true in the high conflict condition. These investigators did not observe any activity in the ACC related to this high conflict condition as compared to the low conflict condition. However, recordings of single cells of the caudal ACC in humans showed graded responses to conflict during the Stroop task, though many seemed related to emotional salience and difficulty [76]. Taken together, the current findings along with these earlier studies suggest that, although the ACC is part of neural circuit that responds to conflict to expedite subsequent prefrontal processes of cognitive control, it does not accomplish the task independently.

### Limitations of the study

First, compared to post-go stop trials, post-stop stop trials activated several prefrontal structures as well as regions in the cerebellar cortex. As theorized by the conflict monitoring hypothesis, the post-conflict activations reflect a process in which these brain regions are engaged in cognitive control. Note that, in studies of the Stroop task, the extent of post-conflict cognitive control could be quantified by post-conflict changes in reaction time. In contrast, in the current study, cognitive control as reflected by the post-conflict lateral prefrontal activation during stop success trials could only be assumed, because stop success trials by definition did not involve a reaction time. Second, the results that the ACC does not have a mediating role in the association between conflict and post-conflict processing need to be considered along with several methodological issues of mediation analyses. As with other methods based on structural equation models, one assumed that all relevant variables are included in the mediation analysis; i.e., one could not rule out the existence of mediating factors not tested in the model [77]. In addition, mediation analysis is only valid upon correct specification of the causal orders [78]. Finally, as pointed out by Wager et al. [79], an additional limitation of using mediation analysis in fMRI is that models are made on the basis of naturally occurring variance over subjects, and thus conclusions are made with the assumption that inter-subject variability does not affect the coupling between dependent variables [79]. Third, the stop signal task and Stroop task may involve fundamentally different neural processes in cognitive control. For instance, while previous studies of the Stoop task emphasized the role of the ACC in conflict processing, we observed both cortical and subcortical conflict-related activations in the SST. Dorsolatereal prefrontal cortex (DLPFC) was implicated in post-conflict control in earlier studies, whereas we observed orbitofrontal and frontopolar in addition to DLPFC activations during post-conflict control in the SST. Thus, although the current results do not provide support for the conflict monitoring theory, we could not rule out the possibility that the discrepancy may simply reflect differences in behavioral tasks. Fourth, the current results were obtained with a relatively liberal threshold. In reporting the correlation results, we used an arbitrary threshold of p<0.005 to highlight the differences between error and non-error processes. These results are thus preliminary and need to be replicated in the future.

### Conclusions

We have two main conclusions to draw from the current results. First, although ACC activity during conflict processing does correlate with prefrontal post-conflict activity, this correlation is not unique to the ACC, in the stop signal task. Second, thalamic and insular but not ACC activity during error processing correlates with prefrontal post-conflict activity, suggesting distinct neural pathways for non-error and error conflict control in the stop signal task.

## References

[1] Botvinick MM, Braver TS, Barch DM, Carter CS, Cohen JD (2001). Conflict monitoring and cognitive control.. Psychol Rev.

[2] Botvinick M, Nystrom L, Fissell K, Carter CS, Cohen JD (1999). Conflict monitoring versus selection-for-action in anterior cingulated cortex.. Nature.

[3] Garavan H, Ross TJ, Murphy K, Roche RAP, Stein EA (2002). Dissociable executive functions in the dynamic control of behavior: Inhibition, error detection, and correction.. NeuroImage.

[4] Ito S, Stuphorn V, Brown JW, Schall JD (2003). Performance monitoring by the anterior cingulate cortex during saccade countermanding.. Science.

[5] Ullsperger M, von Cramon DY (2004). Neuroimaging of performance monitoring: Error detection and beyond.. Cortex.

[6] MacDonald AW, Cohen JD, Stenger VA, Carter CS (2000). Dissociating the role of the dorsolateral prefrontal and anterior cingulate cortex in cognitive control.. Science.

[7] Kerns JG, Cohen JD, MacDonald AW, Cho RY, Stenger VA (2004). Anterior cingulate conflict monitoring and adjustments in control.. Science.

[8] Braver TS, Barch DM, Gray JR, Molfese DL, Snyder A (2001). Anterior cingulate cortex and response conflict: Effects of frequency, inhibition and errors.. Cereb Cortex.

[9] Ullsperger M, von Cramon DY (2001). Subprocesses of performance monitoring: A dissociation of error processing and response competition revealed by event-related fMRI and ERPs.. Neuroimage.

[10] Li CS, Yan P, Chao HH, Sinha R, Paliwal P (2008). Error-specific medial cortical and subcortical activity during the stop signal task: a functional magnetic resonance imaging study.. Neuroscience.

[11] Wittfoth M, Kustermann E, Fahle M, Herrmann M (2008). The influence of response conflict on error processing: Evidence from event-related fMRI.. Brain Res.

[12] Li CS, Huang C, Yan P, Paliwal P, Constable RT (2008). Neural correlates of post-error slowing during a stop signal task: a functional magnetic resonance imaging study.. J Cogn Neurosci.

[13] Logan GD, Cowan WB, Davis KA (1984). On the ability to inhibit simple and choice reaction time responses: a model and a method.. J Exp Psychol Hum Percept Perform.

[14] Li CS, Huang C, Constable RT, Sinha R (2006). Imaging response inhibition in a stop-signal task: neural correlates independent of signal monitoring and post-response processing.. J Neurosci.

[15] Li CS, Chao HH, Lee TW (2009). Neural correlates of speeded as compared with delayed responses in a stop signal task: an indirect analog of risk taking and association with an anxiety trait.. Cereb Cortex.

[16] Levitt H (1970). Transformed up-down methods in psychoacoustics.. J Acoust Soc Am.

[17] De Jong R, Coles MG, Logan GD, Gratton G (1990). In search of the point of no return: the control of response processes.. J Exp Psychol Hum Percept Perform.

[18] Friston KJ, Ashburner J, Frith CD, Polone J-B, Heather JD (1995). Spatial registration and normalization of images.. Hum Brain Mapp.

[19] Ashburner J, Friston KJ (1999). Nonlinear spatial normalization using basis functions.. Hum Brain Mapp.

[20] Friston KJ, Holmes AP, Worsley KJ, Poline J-B, Frith CD (1995). Statistical parametric maps in functional imaging: a general linear approach.. Hum Brain Mapp.

[21] Friston KJ, Josephs O, Zarahn E, Holmes AP, Rouquette S (2000). To smooth or not to smooth? Bias and efficiency in fMRI time-series analysis.. Neuroimage.

[22] Penny W, Holmes AP, Frackowiak (2004). Random-effects analysis.. Human Brain Function.

[23] Duvernoy HM (1999). The Human Brain: Surface, Blood Supply, and Three-Dimensional Sectional Anatomy..

[24] Brett M, Anton J-L, Valabregue R, Poline J-P (2002). Region of interest analysis using an SPM toolbox..

[25] MacKinnon DP, Fairchild AJ, Fritz MS (2007). Mediation analysis.. Annu Rev Psychol.

[26] Maccorquodale K, Meehl PE (1948). On a distinction between hypothetical constructs and intervening variables.. Psychol Rev.

[27] Baron RM, Kenny DA (1986). The moderator-mediator variable distinction in social psychological research: conceptual, strategic, and statistical considerations.. J Pers Soc Psychol.

[28] Wager TD, Davidson ML, Hughes BL, Lindquist MA, Ochsner KN (2008). Prefrontal-subcortical pathways mediating successful emotion regulation.. Neuron.

[29] Botvinick MM, Cohen JD, Carter CS (2004). Conflict monitoring and anterior cingulate cortex: An update.. Trends Cogn Sci.

[30] Gruber O, Goschke T (2004). Executive control emerging from dynamic interactions between brain systems mediating language, working memory and attentional processes.. Acta Psychologica.

[31] Ridderinkhof KR, van den Wildenberg WP, Segalowitz SJ, Carter CS (2004). Neurocognitive mechanisms of cognitive control: the role of prefrontal cortex in action selection, response inhibition, performance monitoring, and reward-based learning.. Brain Cogn.

[32] Carter CS, van Veen V (2007). Anterior cingulate cortex and conflict detection: an update of theory and data.. Cogn Affect Behav Neurosci.

[33] Hajcak G, McDonald N, Simons RF (2003). To err is autonomic: Error-related brain potentials, ANS activity, and post-error compensatory behavior.. Psychophysiology.

[34] Hajcak G, Nieuwenhuis S, Ridderinkhof KR, Simons RF (2005). Error-preceding brain activity: Robustness, temporal dynamics, and boundary conditions.. Biol Psychol.

[35] Brown JW (2008). Multiple cognitive control effects of error likelihood and conflict.. Psychol Res.

[36] Modirrousta M, Fellows LK (2008). Dorsal medial prefrontal cortex plays a necessary role in rapid error prediction in humans.. J Neurosci.

[37] Gehring WJ, Goss B, Coles MGH, Meyer DE, Donchin E (1993). A neural system for error-detection and compensation.. Psychol Sci.

[38] Gehring WJ, Knight RT (2000). Prefrontal-cingulate interactions in action monitoring.. Nat Neurosci.

[39] Bush G, Luu P, Posner MI (2000). Cognitive and emotional influences in anterior cingulate cortex.. Trends Cogn Sci.

[40] Holroyd CB, Coles MG (2002). The neural basis of human error processing: Reinforcement learning, dopamine, and the error-related negativity.. Psychol Rev.

[41] Gehring WJ, Fencsik DE (2001). Functions of the medial frontal cortex in the processing of conflict and errors.. J Neurosci.

[42] Hajcak G, Simons RF (2008). Oops!.. I did it again: An ERP and behavioral study of double-errors.. Brain Cogn.

[43] Dudschig C, Jentzsch I (2009). Speeding before and slowing after errors: Is it all just strategy?. Brain Research.

[44] Debener S, Ullsperger M, Siegel M, Fiehler K, von Cramon DY (2005). Trial-by-trial coupling of concurrent electroencephalogram and functional magnetic resonance imaging identifies the dynamics of performance monitoring.. J Neurosci.

[45] West R, Travers S (2008). Tracking the temporal dynamics of updating cognitive control: an examination of error processing. Cereb.. Cortex.

[46] Ide JS, Li C-SR (In press). A cerebellar thalamic cortical circuit for error-related cognitive control.. Neuroimage.

[47] Diamond ME, Ahissar E (2007). When outgoing and incoming signals meet: new insights from the zona incerta.. Neuron.

[48] Urbain N, Deschênes M (2007). Motor cortex gates vibrissal responses in a thalamocortical projection pathway.. Neuron.

[49] Mitchell AS, Browning PG, Baxter MG (2007). Neurotoxic lesions of the medial mediodorsal nucleus of the thalamus disrupt reinforcer devaluation effects in rhesus monkeys.. J Neurosci.

[50] Wagner G, Koch K, Reichenbach JR, Sauer H, Schlosser RG (2006). The special involvement of the rostrolateral prefrontal cortex in planning abilities: an event-related fMRI study with the Tower of London paradigm.. Neuropsychologia.

[51] Sommer MA, Wurtz RH (2004). What the brain stem tells the frontal cortex. II. Role of the SC-MD-FEF pathway in corollary discharge.. J Neurophysiol.

[52] Bellebaum C, Daum I, Koch B, Schwarz M, Hoffmann KP (2005). The role of the human thalamus in processing corollary discharge.. Brain.

[53] Monchi O, Petrides M, Petre V, Worsley K, Dagher A (2001). Wisconsin Card Sorting revisited: distinct neural circuits participating in different stages of the task identified by event-related functional magnetic resonance imaging.. J Neurosci.

[54] Yamamoto T, Yoshida K, Yoshikawa H, Kishimoto Y, Oka H (1992). The medial dorsal nucleus is one of the thalamic relays of the cerebellocerebral responses to the frontal association cortex in the monkey: horseradish peroxidase and fluorescent dye double staining study.. Brain Res.

[55] Jones EG (2002). Thalamic circuitry and thalamocortical synchrony.. Philos Trans R Soc Lond B Biol Sci.

[56] Stepniewska I, Preuss TM, Kaas JH (2007). Thalamic connections of the dorsal and ventral premotor areas in New World owl monkeys.. Neuroscience.

[57] Fassbender C, Murphy K, Foxe JJ, Wylie GR, Javitt DC (2004). A topography of executive functions and their interactions revealed by functional magnetic resonance imaging.. Cogn Brain Res.

[58] Hester R, Fassbender C, Garavan H (2004). Individual differences in error processing: a review and reanalysis of three event-related fMRI studies using the GO/NOGO task.. Cereb Cortex.

[59] Magno E, Foxe JJ, Molholm S, Robertson I, Garavan H (2006). The anterior cingulate and error avoidance.. J Neurosci.

[60] Braet W, Johnson KA, Tobin CT, Acheson R, McDonnell C (2009). Increased fMRI activation during response inhibition, and decreased activation during error processing is associated with possession of the 10-repeat allele of the DAT1 gene: a genetic imaging study investigating the role of the DAT1 gene in Attention Deficit Hyperactivity disorder.. Neuroimage.

[61] Ramautar JR, Slagter HA, Kok A, Ridderinkhof KR (2006). Probability effects in the stop-signal paradigm: The insula and the significance of failed inhibition.. Brain Research.

[62] Eckert MA, Menon V, Walczak A, Ahlstrom J, Denslow S (2009). At the heart of the ventral attention system: the right anterior insula.. Hum Brain Mapp.

[63] Critchley HD, Corfield D, Chandler M, Mathias CJ, Dolan RJ (2000). Cerebral correlates of peripheral cardiovascular arousal: a functional neuroimaging study.. J Physiol.

[64] Critchley HD, Mathias CJ, Dolan RJ (2002). Fear-conditioning in humans: the influence of awareness and arousal on functional neuroanatomy.. Neuron.

[65] Cameron OG, Minoshima S (2002). Regional brain activation due to pharmacologically induced adrenergic interoceptive stimulation in humans.. Psychosom Med.

[66] Ploran EJ, Nelson SM, Velanova K, Donaldson DI, Petersen SE (2007). Evidence accumulation and the moment of recognition: dissociating perceptual recognition processes using fMRI.. J Neurosci.

[67] Vendrell P, Junque C, Pujol J, Jurado MA, Molet J (1995). The role of prefrontal regions in the Stroop task.. Neuropsychologia.

[68] Stuss DT, Floden D, Alexander MP, Levine B, Katz D (2001). Stroop performance in focal lesion patients: dissociation of processes and frontal lobe lesion location.. Neuropsychologia.

[69] Erickson KI, Milham MP, Colcombe SJ, Kramer AF, Banich MT (2004). Behavioral conflict, anterior cingulate cortex, and experiment duration: implications of diverging data.. Hum Brain Mapp.

[70] Fellows LK, Farah MJ (2005). Different underlying impairments in decision-making following ventromedial and dorsolateral frontal lobe damage in humans.. Cereb Cortex.

[71] Baird A, Dewar BK, Critchley H, Gilbert SJ, Dolan RJ (2006). Cognitive functioning after medial frontal lobe damage including the anterior cingulate cortex: a preliminary investigation.. Brain Cogn.

[72] Roelofs A, van Turennout M, Coles MG (2006). Anterior cingulate cortex activity can be independent of response conflict in Stroop-like tasks.. Proc Natl Acad Sci.

[73] Mansouri FA, Tanaka K, Buckley MJ (2009). Conflict-induced behavioural adjustment: a clue to the executive functions of the prefrontal cortex.. Nat Rev Neurosci.

[74] Mansouri FA, Buckley MJ, Tanaka K (2007). Mnemonic function of the dorsolateral prefrontal cortex in conflict-induced behavioral adjustment.. Science.

[75] Nakamura K, Roesch MR, Olson CR (2005). Neuronal activity in macaque SEF and ACC during performance of tasks involving conflict.. J Neurophysiol.

[76] Davis KD, Taylor KS, Hutchison WD, Dostrovsky JO, McAndrews MP (2005). Human anterior cingulate cortex neurons encode cognitive and emotional demands.. J Neurosci.

[77] Lebrecht S, Badre D (2008). Emotional regulation, or: how I learned to stop worrying and love the nucleus accumbens.. Neuron.

[78] MacKinnon DP, Fairchild AJ, Fritz MS (2007). Mediation analysis.. Annu Rev Psychol.

[79] Wager TD, Davidson ML, Hughes BL, Lindquist MA, Ochsner KN (2008). Prefrontal-subcortical pathways mediating successful emotion regulation.. Neuron.

